# Physiological status and anatomical severity factors associated with child versus adult bicyclist fatalities based on a national trauma dataset

**DOI:** 10.1038/s41598-022-21949-z

**Published:** 2022-11-01

**Authors:** Wataru Ishii, Masahito Hitosugi, Kenji Kandori, Michitaro Miyaguni, Ryoji Iizuka

**Affiliations:** 1 Critical Care Center, Emergency Medicine, Kyoto Daini Red Cross Hospital, Haruobi, Kamazamarutamachi, Kamigyo, Kyoto 602-8026 Japan; 2 Department of Legal Medicine, Shiga University of Medical Science, Tsukinowa, Seta, Otsu, Shiga 520-2192 Japan

**Keywords:** Epidemiology, Paediatric research, Disease prevention, Trauma

## Abstract

Bicyclists still account for the majority of child deaths in traffic accidents, despite a gradual decrease in incidence. Therefore, we investigated factors associated with child and adult bicyclist fatalities. In this retrospective study, we used data from a national hospital-based database, the Japan Trauma Data Bank. Data from 2004 to 2019 were obtained for child cyclists (5–18 years; n = 4832) and adult cyclists (26–45 years; n = 3449). In each age group, physiological variables, outcomes, and injury severity were compared between fatal and non-fatal cases. Multivariate logistic regression was performed to determine factors associated with fatality. In adults, fatality was associated with lower values for body temperature, Glasgow Coma Scale score, and Abbreviated Injury Scale (AIS) score for the neck and upper extremities, and with higher values for respiratory rate, heart rate, focused assessment with sonography for trauma positivity rate, and AIS scores for the head, chest, and abdomen. In children, fatality was associated with lower values for body temperature and the Glasgow Coma Scale score, and with higher values for the AIS chest score. These findings point to factors associated with bicyclist fatalities and may help in the development of effective strategies to reduce these fatalities.

## Introduction

More than 40,000 bicyclists worldwide are killed every year in bicycle accidents^1^. Although the number of cyclist fatalities is slowly decreasing, it is still high compared with the declining rate of motor vehicle-related fatalities^2^. At present, fatalities due to road collisions by age group in Japan remain high, and 27.1% of road collision fatalities occur in people younger than 19 years.

In Japan, the population aged 5 to 19 years has decreased from 26.0 million in 1990 to 16.2 million in 2020^3^. In 2020, there were 841,000 births and 1.51 million children under the age of 15, which are the lowest numbers ever reported^4^. Therefore, protecting children from road traffic collisions has become a high priority in Japan.

The Japanese government enacted the Basic Law on Traffic Safety Measures in 1970^5^. Under this law, targets have been set in the Basic Plan for Traffic Safety every 5 years since 1971, and traffic safety measures have been comprehensively and systematically promoted. The original goal of these efforts was to realize a society without motor vehicle collisions. In 2021, the Japanese government set a new target that aims to reduce the number of road-related fatalities to less than 2,000 and the number of serious injuries to less than 22,000 by 2025^6^. In addition, as the child population continues to decline, improving the environment so that children can be safe while they are active and growing is also mentioned in this policy. Because riding bicycles is popular among children, preventing child bicyclist fatalities should be promoted. With regard to bicycle accidents among young cyclists, previous studies have investigated environmental factors (behavioral patterns, regional characteristics) and trauma patterns of bicycle accidents in an effort to prevent cyclist accidents^7–9^. To identify effective preventive measures, factors that influence child bicyclist fatalities need to be clarified. However, no studies have investigated the factors that influence bicyclist fatalities in Japan. Therefore, the objective of this study was to clarify the physiological characteristics and metrics related to injury severity in child bicyclist fatalities and compare them with those of young adults. We also focused on child bicyclists who were transferred to an emergency hospital. In Japan, to help standardize emergency care, physiological parameters and anatomical injury severities are assessed in an initial examination. These data were used to identify factors that influence bicyclist fatalities.

## Materials and methods

### Study design and patient selection

This observational study was a retrospective analysis of data from a national hospital-based database called the Japan Trauma Data Bank (JTDB). The JTDB is a national trauma registry in Japan that includes data recorded by the Japanese Society of Trauma Surgeons and the Japanese Society of Emergency Medicine and has been in existence since 2003. This registry is similar to trauma databases in North America, Europe, and Oceania^10–13^. Approximately 372,000 trauma patients have been registered as of 2019^10^. In 2005, 55 hospitals participated in the registry; in 2019, more than 288 hospitals participated in the registry, which accounted for approximately 75% of all emergency centers in Japan. Although the number of registered hospitals has increased over the years, the percentage of emergency centers among these has not changed. The JTDB collects information on the mode and mechanism of trauma, vital signs, the anatomical and physiological severity of injury, pre-hospital and in-hospital treatment, and outcomes.

**Figure 1:**
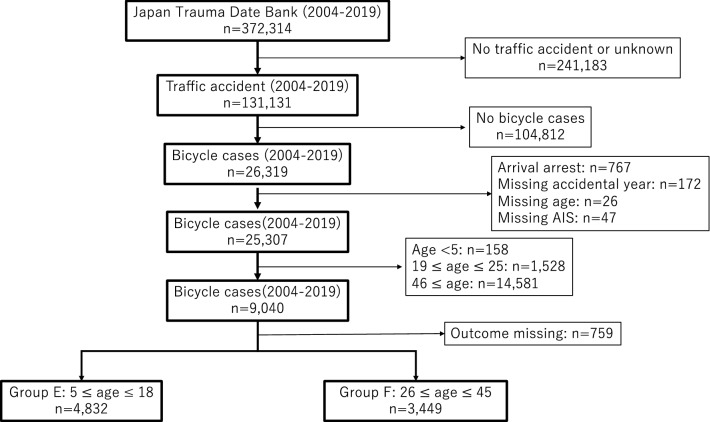
Flowchart of patient enrolment.

The data were obtained from the JTDB in December 2020. A total of 372,314 patients were registered in the JTDB from 2004 to 2019. Of these, 26,319 were patients with bicycle collision-related trauma. We excluded patients if they arrived at the hospital with cardiopulmonary arrest (n = 767), if their age, accident year, or Abbreviated Injury Scale score was unclear, or if they had missing data (n = 245). After applying the exclusion criteria, there were 25,307 patients with bicycle-related accident data. Of these, patients between 26 and 45 years old and between 5 and 18 years old were selected, and patients with unknown outcomes (n = 759) were excluded. Finally, 8281 patients were included in the analysis (4832 children and 3449 adults) (Fig. 1).

The following information was obtained for each patient: age, gender, vital signs upon hospital arrival (systolic and diastolic blood pressure, heart rate (HR), respiratory rate (RR), body temperature (BT), and Glasgow Coma Scale (GCS) score), focused assessment with sonography for trauma (FAST) test results, Abbreviated Injury Scale (AIS) scores for each body part (1998 version), Injury Severity Score (ISS), Revised Trauma Score (RTS), Trauma and Injury Severity Score Probability of Survival (TRISSPS), and post-discharge outcome. The AIS score is used to classify the type and severity of injury in each body part on an anatomical scale of 1 (minor) to 6 (clinically untreatable). The ISS is used for assessing the severity of multiple injuries and is the sum of the squares of the highest AIS scores for each of the three most severely injured body parts. Because these scores correlate well with mortality, they are usually assessed as a measure of anatomic severity in an emergency medical setting; in Japan they are evaluated by medical staff who have participated in coding seminars. The RTS is a method of assessing severity based on physiological indices, with the highest score (least severe) being 7.84 and the lowest score (most severe) being 0. The TRISSPS sums the physiological and anatomical severity score of the patient’s condition with their age to calculate a predicted survival (Ps) rate as follows: (1) preventable death (Ps > 0.50), (2) possibly preventable death (0.25 < 0.50), and (3) non-preventable death (Ps < 0.25).

The data in JTDB are based on data from hospitals. The JTDB does not include data from the police department regarding the accident and road conditions. Some studies have been conducted that have used police data, but these studies are rare in Japan. Owing to the lack of police crash data and other data, we decided not to include deaths at the time of the accident in the analysis.

### Statistical analysis

Categorical variables are shown as the proportion or frequency. Continuous variables are shown as the mean ± standard deviation for values that followed a normal distribution and as the median and interquartile range for values that were not normally distributed. The chi-square test was used to compare the prevalence between two groups. To identify differences in values between two groups, Student’s t-test was used for values with a normal distribution, while the Wilcoxon test was used for values with a non-normal distribution. A p-value of ≤ 0.05 was considered statistically significant. Logistic regression analyses were performed to identify variables that were independently associated with a poor outcome or having severe injury. The analyses were performed with JMP pro15.2 (SAS Institute Inc., 2020.).

### Endpoint

We examined the factors that were significantly associated with child bicyclist fatalities using data from the JTDB.

### Ethics

Personal identifiers had already been removed from the JTDB. This study was conducted in accordance with the Declaration of Helsinki and approved by the Ethical Committee of Kyoto Daini Red Cross Hospital (Sp2022-02). The need for informed consent was waived by the ethics committee that approved the study protocol because of the anonymous and retrospective nature of this study.

## Results

### Outcomes of adult bicyclists (26–45 years old)

In the adult bicyclist group, 146 (4.2%) patients died (fatal group) and 3303 survived (non-fatal group). The patient background characteristics and vital signs upon hospital arrival were compared between the two groups (Table 1). Compared with the non-fatal group, the fatal group had a significantly lower BT and GCS score and a significantly higher HR, RR, and FAST positivity rate upon hospital arrival. Regarding injury severity, the head, chest, and abdominal AIS scores and the ISS score were significantly higher, and the neck and upper extremities AIS scores were significantly lower in the fatal group than in the non-fatal group (Table 2). The RTS and TRISSPS scores were significantly lower in the fatal group than in the non-fatal group.

**Table 1:** Patient characteristics and data upon hospital arrival for adults (26–45 years) in the fatal and non-fatal groups (2004–2019).

	Non-fatal (n = 3303)	Fatal (n = 146)	P value
Age (years)	36.3 ± 5.8	36.3 ± 5.8	0.9421
**Sex, n (%)**			
Male	67.6	67.8	0.951
Female	32.4	32.2	
Systolic blood pressure (mmHg)	129.7 ± 23.2	127.4 ± 42.2	0.7192
Diastolic blood pressure (mmHg)	79.3 ± 17.0	81.2 ± 25.8	0.3391
Heart rate (beats/min)	83.2 ± 17.3	92.8 ± 31.6	0.0174
Respiration rate (breaths/min)	21.0 ± 6.5	23.1 ± 10.6	0.0002
Body temperature (℃)	36.5 ± 0.7	36.0 ± 0.9	< 0.0001
Glasgow coma scale	13.6 ± 2.7	6.0 ± 3.7	< 0.0001
FAST positive (%)	4.4	13.7	< 0.0001

*FAST* focused assessment with sonography for trauma.

Student’s t-test was used for values with a normal distribution, while the Wilcoxon test was used for values with a non-normal distribution.

**Table 2:** AIS score by body region, ISS, RTS, and TRISSPS score in adults (26–45 years) in the fatal and non-fatal groups (2004–2019).

	Non-fatal (n = 3303)	Fatal (n = 146)	P value
**AIS, median (IQR)**			
Head**	1.0 (0.0–3.0)	5.0 (4.0–5.0)	< 0.0001
Face	0.0 (0.0–0.0)	0.0 (0.0–0.0)	0.2180
Neck*	0.0 (0.0–1.0)	0.0 (0.0–0.0)	0.0178
Chest**	0.0 (0.0–1.0)	1.0 (0.0–4.0)	< 0.0001
Abdomen **	0.0 (0.0–0.0)	0.0 (0.0–0.0)	0.0013
Spine	0.0 (0.0–0.0)	0.0 (0.0–0.0)	0.6210
Upper extremities*	0.0 (0.0–1.0)	0.0 (0.0–0.0)	0.0005
Lower extremities	0.0 (0.0–1.0)	0.0 (0.0–1.0)	0.3241
ISS, median (IQR)**	13.0 (9.0–19.0)	29.0 (25.0–41.0)	< 0.0001
RTS, median (IQR)*	7.84 (7.55–7.84)	4.89 (4.09–5.97)	< 0.0001
TRISSPs, median (IQR)*	0.99 (0.98–0.99)	0.68 (0.44–0.89)	< 0.0001

*AIS* abbreviated injury scale, *ISS* injury severity score, *IQR* interquartile range, *RTS* revised trauma scale, *TRISSPS* trauma and injury severity score probability of survival.

*The median value in the non-fatal group was significantly higher than that in the fatal group.

**The median value in the non-fatal group was significantly lower than that in the fatal group.

Student’s t-test was used for values with a normal distribution, while the Wilcoxon test was used for values with a non-normal distribution.

### Outcomes of child bicyclists (5–18 years old)

In the child bicyclist group, 96 patients (2.0%) died (fatal group) and 4736 survived (non-fatal group). The patient background characteristics and vital signs upon hospital arrival were compared between the two groups (Table 3). Compared with the non-fatal group, the fatal group had a significantly lower BT and GCS score and a significantly higher age and FAST positivity rate upon hospital arrival. Regarding injury severity, the head, chest, and spine AIS scores and the ISS score were significantly higher, and the upper extremity AIS score was significantly lower in the non-fatal group than in the fatal group (Table 4). The RTS and TRISSPS scores were significantly lower in the fatal group than in the non-fatal group.

**Table 3:** Patient characteristics and data upon hospital arrival for children (5–18 years) in the fatal and non-fatal groups (2004–2019).

	Non-fatal (n = 4736)	Fatal (n = 96)	P value
Age (years)	12.8 ± 3.7	13.8 ± 3.4	0.0042
**Sex, n (%)**			
Male	69.7	69.8	0.9606
Female	30.2	30.2	
Systolic blood pressure (mmHg)	125.7 ± 18.5	121.3 ± 40.7	0.5398
Diastolic blood pressure (mmHg)	72.6 ± 14.7	73.5 ± 24.2	0.9785
Heart rate (beats/min)	92.0 ± 20.0	99.4 ± 34.6	0.3126
Respiration rate (breaths/min)	22.2 ± 6.4	21.7 ± 10.5	0.6718
Body temperature (℃)	36.7 ± 0.7	36.1 ± 1.2	< 0.0001
Glasgow coma scale	13.4 ± 2.8	5.0 ± 3.2	< 0.0001
FAST positive (%)	4.8	11.5	0.0206

*FAST* focused assessment with sonography for trauma.

Student’s t-test was used for values with a normal distribution, while the Wilcoxon test was used for values with a non-normal distribution.

**Table 4:** AIS score by body region, ISS, RTS, and TRISSPS in children (5–18 years) in the fatal and non-fatal groups (2004–2019).

	Non-fatal (n = 4736)	Fatal (n = 96)	P value
**AIS, median (IQR)**			
Head **	2.0 (0.0–4.0)	5.0 (4.0–5.0)	< 0.0001
Face	0.0 (0.0–0.0)	0.0 (0.0–0.0)	0.8153
Neck	0.0 (0.0–1.0)	0.0 (0.0–0.0)	0.2109
Chest **	0.0 (0.0–0.0)	3.0 (0.0–4.0)	< 0.0001
Abdomen	0.0 (0.0–0.0)	0.0 (0.0–0.0)	0.1846
Spine **	0.0 (0.0–0.0)	0.0 (0.0–0.0)	0.0106
Upper extremities *	0.0 (0.0–1.0)	0.0 (0.0–0.0)	0.0043
Lower extremities	0.0 (0.0–1.0)	0.0 (0.0–2.0)	0.6343
ISS, median (IQR)**	10.0 (8.0–17.0)	32.0 (25.0–42.0)	< 0.0001
RTS, median (IQR)*	7.84 (7.55–7.84)	4.09 (3.51–5.35)	< 0.0001
TRISSPs, median (IQR)*	0.99 (0.98–0.99)	0.61 (0.29–0.82)	< 0.0001

*AIS* abbreviated injury scale, *ISS* injury severity score, *IQR* interquartile range, *RTS* revised trauma scale, *TRISSPS* trauma and injury severity score probability of survival.

*The median value in the non-fatal group was significantly higher than that in the fatal group.

**The median value in the non-fatal group was significantly lower than that in the fatal group.

Student’s t-test was used for values with a normal distribution, while the Wilcoxon test was used for values with a non-normal distribution.

### Factors associated with outcomes in adults (26–45 years old)

To identify variables that were independently associated with fatality, logistic regression analysis was performed using the HR, RR, BT, GCS score, FAST positivity rate, and AIS scores for head, neck, chest, abdomen, upper extremities, and lower extremities as independent variables. The following factors were identified as independent predictors of a fatal outcome: RR (odds ratio [OR] [95% confidence Interval]: 1.05 [1.02–1.09]), HR (OR: 1.01 [1.00–1.02]), BT (OR: 0.63 [0.47–0.85]), GCS score (OR: 0.68 [0.63–0.72]), FAST positivity rate (OR: 4.07 [1.64–10.11]), and the AIS scores for head (OR: 1.59 [1.32–1.93]), neck (OR: 0.66 [0.45–0.95]), chest (OR: 1.36 [1.17–1.58]), and upper extremities (OR: 0.60 [0.40–0.87]) (Table 5).

**Table 5:** Multivariate analysis results of factors associated with adult outcomes (26–45 years).

(n = 3449)	OR	95% confidence interval	P-value
RR	1.05	1.02–1.09	0.0010
HR	1.01	1.00–1.02	0.0164
BT	0.63	0.47–0.85	0.0021
GCS	0.68	0.63–0.72	< 0.0001
FAST	4.07	1.64–10.11	0.0025
AIS head	1.59	1.32–1.93	< 0.0001
AIS neck	0.66	0.45–0.95	0.0230
AIS chest	1.36	1.17–1.58	< 0.0001
AIS abdomen	1.31	0.97–1.74	0.0735
AIS upper extremities	0.60	0.40–0.87	0.0063
AIS lower extremities	1.11	0.89–1.38	0.3386

*AIS* abbreviated injury scale, *BT* body temperature, *CI* confidence interval, *FAST* focused assessment with sonography for trauma, *GCS* Glasgow Coma Scale, *HR* heart rate, *OR* odds ratio, *RR* respiratory rate.

Logistic regression analyses.

### Factors associated with outcomes in children (5–18 years old)

To identify variables independently associated with fatality, logistic regression analysis was performed for both adults and children using the HR, RR, BT, GCS score, FAST positivity rate, and AIS scores for head, neck, chest, abdomen, upper extremities, and lower extremities as independent variables. The data were subjected to regression analysis. BT (OR: 0.69 [0.50–0.94]), the GCS score (OR: 0.58 [0.52–0.65]), and the AIS score for chest (OR: 1.39 [1.15–1.67]) were identified as independent predictors of a fatal outcome (Table 6).

**Table 6:** Multivariate analysis results of factors associated with child outcomes (5–18 years).

(n = 4832)	OR	95% confidence interval	P-value
RR	1.01	0.97–1.05	0.6220
HR	1.00	0.99–1.01	0.7058
BT	0.69	0.50–0.94	0.0228
GCS	0.58	0.52–0.65	< 0.0001
FAST	1.08	0.27–4.30	0.9184
AIS head	1.12	0.86–1.45	0.4055
AIS neck	0.98	0.63–1.51	0.9203
AIS chest	1.39	1.15–1.67	0.0007
AIS abdomen	1.01	0.71–1.45	0.9498
AIS upper extremities	0.78	0.97–1.05	0.2759
AIS lower extremities	1.01	0.76–1.35	0.9316

*AIS* abbreviated injury scale, *BT* body temperature, *CI* confidence interval, *GCS* Glasgow Coma Scale, *OR* odds ratio.

Logistic regression analyses.

## Discussion

According to the 2020 statistics in Japan, 20.6% of the fatalities and serious injuries in traffic accidents were pedestrians, 18.8% were motorcyclists, and 10.4% were cyclists^14^. However, bicycling was the second leading cause of traffic fatalities among elementary school students from 2011 to 2020, accounting for 34.9% of all traffic-related fatalities in this group. In addition, while overall traffic fatalities decreased by 31% overall between 2015 and 2020, the decreases in motorcycle- and bicycle-related fatalities were smaller^14^.

Monitoring trauma trends and outcomes can help identify effective injury prevention strategies and treatments. The JTDB registers data on trauma patients and records pre-hospital and hospital-related information, including clinical outcomes. Thus, analyses using information from this database can provide evidence to support injury prevention measures. Three previous reports have examined child injuries using JTDB data^15–17^. One of the studies focused on child injuries from motor vehicle collisions and described injury patterns and outcomes related to the seating position of the child within the vehicle. The study suggested that for children aged 6–12 years, the incidences of head and chest injuries were higher in the rear seats than in the front seats. However, no studies using the JTDB have examined factors that are independently associated with bicyclist fatalities.

Here, we aimed to clarify factors that are associated with fatalities in children and adult bicyclists, with the adult group being defined as those aged 26–45 years old. Because age is significantly associated with injury severity owing to decreasing physical robustness and the increasing prevalence of comorbidities with age, we excluded cyclists older than 45 years. We established the child group as those aged 5–18 years because the milestone of learning to ride a bicycle often occurs around 5 years of age^18^.

According to a report from Australia, cyclists involved in traffic-related accidents frequently suffered multiple injuries (38.8%), and the injured sites included the head (31.3%), chest (4.8%), neck (3.4%), and brain (2.0%)^19^. Riding a bicycle requires neurological integration of motor, sensory, visual, and balance skills^20^. Children initially concentrate on balancing while moving in a straight line^21^, and they can have difficulty integrating the central vision needed for balance with the peripheral vision needed to detect traffic hazards. These developmental considerations affect children’s neurophysiological readiness and ability to travel safely on roads in the presence of other vehicles^18^. It is important to improve children’s environment by allowing younger children to ride bicycles on sidewalks. Roads should be designed to separate bicycles from automobiles, especially for child cyclists.

Both children and adult bicyclist fatalities had lower values of BT and GCS scores and a higher FAST positivity rate compared with nonfatalities. These findings are in agreement with previous reports that examined the factors influencing motor vehicle collision fatalities^17,22^. The RR and HR were significantly higher in the fatal group than the nonfatal group, but only in adults. Because the baseline of these values was higher in children than in adults, no significant differences were observed in children between these variables. Regarding injury severity, the ISS, RTS, and TRISSPS score were significantly higher in the fatal group than in the nonfatal group for both children and adults, and similar values were obtained for children and adults. However, there were some differences concerning the injured body regions. In adults, the head, chest and abdomen AIS values were significantly higher in the fatal group than in the nonfatal group. In children, the head, chest and spine AIS scores were significantly higher in the fatal group than in the nonfatal group. Subsequently, according to the logistic regression analyses, the AIS values for the head, and chest were positively associated with fatality in adults, whereas only the AIS chest value was positively associated with fatality in children. These differences might be owing to differences between adults and children regarding the circumstances of collisions and the features and mechanisms of injuries. First, although the head is the most commonly injured body region in adult and child bicyclists^19,23–27^, children survive head injuries more often than adults. The adult brain is considered to be less amenable to physiological and neuroanatomical reorganization following injury^28,29^. Compared with adults, better functional outcomes following traumatic brain injury have been observed in children^30,31^. This might be why the AIS head value was not associated with fatality in children. Second, adults cycle for transportation, leisure, and sport, and children tend to ride their bikes in residential areas and side streets and at lower velocity^32^. Therefore, the relative velocity might be lower in collisions involving children. Third, owing to their lower body weight, children are more likely to be thrown in a collision. For this reason, in addition to the differences in body size, the prevalence of injured body regions in adults and children would differ. When thrown during a collision, because of their larger proportion of chest area, children might more often suffer from chest injuries than abdominal injuries. This might be why the abdomen AIS score and the FAST positivity rate were not significantly associated with fatality in children. These findings might be useful to help establish further preventive measures.

To protect children from trauma, it may be advisable for them to wear a vest that protects the chest^26,33,34^. Although there is no legislation requiring bicyclists to wear a chest protector in Japan, the development and widespread use of chest protectors would be beneficial. Regarding helmet use, bicyclists younger than 13 years of age are required to wear a helmet according to Japanese road traffic regulations; however, this is not enforced. We recommend that local governments promote the wearing of helmets to improve their use in Japan.

We found that the GCS score and BT were negatively associated with child bicyclist fatality and that the AIS chest value was positively associated with child bicyclist fatality. The GCS score, BT, and AIS chest value have been previously determined to be independent predictors of fatality for child passengers involved in motor vehicle accidents^17^. The GCS score and BT are similar indices that are used to assess the body’s condition, but the injured body regions differ between vehicle passengers and bicyclists. Because the mechanisms of injury differ according to the manner of collision, physicians should consider whether the victim was a cyclist or a motor vehicle passenger when investigating the injury severity.

## Conclusion

The physiological parameters and injury characteristics associated with bicyclist fatalities among children and adults were determined. Because these data cannot be obtained through national accident statistics or police reports, our results provide valuable information for the further prevention of fatalities from a medical viewpoint. Our study suggests the need for patients to be transported to a facility handling critical care and early treatment intervention when particular injury characteristics are observed. Additionally, our findings are useful for practical emergency physicians caring for both adult and child bicyclists, indicating that intensive care should be started as soon as possible if physiological or anatomical parameters that may cause fatality are observed. However, further research integrating medical data with police data including the circumstances of collisions is required to confirm the present results.

## Limitations

A limitation of this study was that it did not include all injured cyclists in Japan. However, the JTDB represents trauma cases to a similar degree as corresponding databases in North America, Europe, and Oceania. In addition, almost all certified trauma educational institutions and many critical care centers contribute data to this database. Because the JTDB is the only prospective, nationwide, hospital-based trauma registry, we believe our analyses provided representative results. Second, information on crashes, such as collision details (type of bicycle, collision direction, and velocity) and helmet use are not included in the JTDB registry. Helmets are not mandatory for adult bicycle riders in Japan and most people do not wear them, so the prevalence of helmet use had little impact on the study outcomes. Further studies could examine the relationships between accident data collected by the police and the medical data collected from hospital records in Japan. Regarding helmet use, according to the Japan Road Traffic Law, child bicyclists younger than 13 years are required to wear a helmet, but this is not enforced with any penalties. As most bicyclists in Japan do not wear them, the prevalence of helmet use had little impact on the study outcomes. Ongoing efforts to improve the helmet use rate should be promoted by local governments in Japan. Relatedly, care is needed when comparing the patterns or severities of head injuries of bicyclists in Japan with those in other countries in which helmet use is legally required, such as Australia and some states in the US. Third, there were informational flaws related to the JTDB dataset. In the JTDB, numerical values or other applicable items are registered via the website, but written information cannot be entered. Therefore, some of the items had missing data. However, because unclear and missing data were excluded from the analysis, the analyzed data had higher reliability. Fourth, as this database was hospital-based, fatalities that occurred at the site of the collision were not included, and we considered that including these out-of-hospital deaths would not improve the reliability of the analyses. Furthermore, patients who exhibited cardiopulmonary arrest upon hospital arrival were excluded from this study. In these cases, the physiological parameters and injury details were not obtained because the examinations were not carried out. Fifth, the JTDB does not state the location of registered hospitals, making it difficult to include the day of the week and time of day of an injury. Insurance information was also not included in the data; however, because health insurance is taken out by nearly all Japanese citizens, its ownership status by a patient was not considered to affect the analysis outcomes. Because of the absence of police crash data and other data, we did not include deaths at the time of the accident in our analysis.

## Data Availability

The data that support the findings of this study are available from Japan Trauma Care and Research but restrictions apply to the availability of these data, which were used under license for the current study, and so are not publicly available. Data are however available from the authors upon reasonable request and with permission of Japan Trauma Care and Research.

## References

[1] Cyclist safety: an information resource for decision-makers and practitioners - World Health Organization, https://www.roadsafetyfacility.org/publications/cyclist-safety-information-resource-decision-makers-and-practitioners (2021–11–15).

[2] National police agency in Japan website, https://www.npa.go.jp/publications/statistics/koutsuu/jiko/R02bunseki.pdf (2021–11–16).

[3] Statistics of Japan; e-Stat is a Portal Site for Japanese Government Statistics, https://www.stat.go.jp/data/jinsui/pdf/202010.pdf (2021-11-20).

[4] Ministry of Health. Labour and Welfare, https://www.mhlw.go.jp/toukei/saikin/hw/jinkou/geppo/nengai20/index.html (2021–11–20).

[5] Traffic Safety Measures in Japan, https://www.mlit.go.jp/road/management-e/e_pdf/0403_5.pdf (2021–11–20).

[6] Cabinet Office, Government of Japan. Basic Traffic Safety Plan, https://www8.cao.go.jp/koutu/kihon/keikaku11/pdf/kihon_keikaku.pdf (2021–11–20).

[7] Katherine WM, Stephen JM, David CL, Andrew R, Charles D (2017). Pediatric emergency department visits for pedestrian and bicyclist injuries in the US. Inj. Epidemiol..

[8] Elizabeth W, Jessica H, Monica S, Manish T, Charles D, Hersch LP, Spiros GF (2017). Urban bicyclist trauma: Characterizing the injuries, consequent surgeries, and essential sub-specialties providing care. Am. Surg..

[9] Pitt TM, Nettel-Aguirre A, McCormack GR, Howard AW, Piatkowski C, Rowe BH, Hagel BE (2019). Child and adolescent bicycling injuries involving motor vehicle collisions. Inj. Epidemiol..

[10] Abe, T., Oda, J., Kimura, A., Sakamoto, Y., Shiraishi, A., Tanaka, K., Tohira, H., Nakahara, S., Hayashi, M., Masuno, T., Miyake, Y., Yamaguchi, Y., Shimizu, K. & Aoki, M. Japan Trauma Date Bank Report 2020, https://www.jtcr-jatec.org/traumabank/dataroom/data/JTDB2020e.pdf (2021-11-21).

[11] Japan Trauma Care and Research. Japan Trauma Data Bank Annual Report 2011–2015, http//www.jtcrjatec.org/traumabank/dataroom/date/JTDB2016.pdf (2021–11–21).

[12] Yumoto T, Mitsuhashi T, Yamakawa Y, Iida A, Nosaka N, Tsukahara K, Naito H, Nakao A (2016). Impact of Cushing’s sign in the prehospital setting on predicting the need for immediate neurosurgical intervention in trauma patients: A nationwide retrospective observational study. Scand. J. Trauma. Resusc. Emerg..

[13] Japan Trauma Care and Research. Japan Trauma Datebank, https//www.jtcr-jatec.org/traumabank/index.htm (2021–11–21).

[14] Traffic safety white paper of Japan in 2021, https://www.shugiin.go.jp/internet/itdb_gian.nsf/html/gian/gian_hokoku/20210831kotsuanzengaiyo.pdf/$File/20210831kotsuanzengaiyo.pdf. (2022–03–09).

[15] Toida C, Muguruma T, Gakumazawa M (2020). Ten-year in-hospital mortality trends among paediatric injured patients in Japan: A nationwide observational study. J. Clin. Med..

[16] Takahashi H, Fujita T, Nakahara S, Sakamoto T (2018). Seating position and patterns of severely injured body parts among child passengers in motor vehicle crashes: Japan as a distinct case. Int. J. Inj. Contr. Saf. Promot..

[17] Ishii W, Hitosugi M, Baba M, Kandori K, Arai Y (2021). Factors affecting death and severe injury in child motor vehicle passengers. Healthcare..

[18] Lenton S, Finlay FO (2018). Public health approaches to safer cycling for children based on developmental and physiological readiness: Implications for practice. BMJ. Paediatr. Open..

[19] Steve OH, Jennie O (2018). Fatal cyclist crashes in Australia. Traffic. Inj. Prev..

[20] Clendon J. Submission to the NZ Transport and Industrial Relations Committee: proposed law change to allow cycling on the footpath (2016).

[21] Clark JE (2007). On the problem of motor skill development. J. Phys. Educ. Recreat. Dance..

[22] Ishii W, Hitosugi M, Takeda A, Baba M, Iizuka R (2020). Factors influencing vehicle passenger fatality have changed over 10 years: A nationwide hospital-based study. Sci. Rep..

[23] Husham, A., Ayman, E. M., Brijesh, S., Rafael, C., Ismail, M., Mohammed, E. & Hassan, A. T. Bicycle-related traumatic injury hospitalizations: six years descriptive analysis in Qatar. *J. Inj. Violence. Res.* **11**, 233–242 (2019). 10.5249/jivr.v11i2.1162 PMC6646826 31280275

[24] de Guerre LEVM, Sadiqi S, Leenen LPH, Oner CF, van Gaalen SM (2020). Injuries related to bicycle accidents: an epidemiological study in The Netherlands. Eur. J. Trauma. Emerg. Surg..

[25] Shams VS, Rajaei GR, Razavi S, Mazouchian H (2016). Bicycle-related injuries presenting to Tabriz Imam Reza Hospital Iran. Trauma. Mon..

[26] Bamini G, Jagnoor J, Ashley C, Annette K, Michael D, Rebecca I, Soufiane B, Ian DC (2016). Describing and comparing the characteristics of injured bicyclists and other injured road users: A prospective cohort study. BMC Public Health.

[27] Teisch LF, Allen CJ, Tashiro J, Golpanian S, Lasko D, Namias N, Neville HL, Sola JE (2015). Injury patterns and outcomes following pediatric bicycle accidents. Pediatr. Surg. Int..

[28] Burda JE, Sofroniew MV (2014). Reactive gliosis and the multicellular response to CNS damage and disease. Neuron.

[29] Nahmani M, Turrigiano GG (2014). Adult cortical plasticity following injury: Recapitulation of critical period mechanisms?. Neuroscience.

[30] Luerssen TG, Klauber MR, Marshall LF (1988). Outcome from head injury related to patient’age: A longitudinal prospective study of adult and pediatric head injury. J. Neurosurg..

[31] Emami P, Czorlich P, Fritzsche FS, Westphal M, Rueger JM, Lefering R, Hoffmann M (2017). Impact of Glasgow Coma Scale score and pupil parameters on mortality rate and outcome in pediatric and adult severe traumatic brain injury: A retrospective, multicenter cohort study. J. Neurosurg..

[32] Siman-Tov, M., Jaffe, D. H., Israel Trauma Group & Peleg K. Bicycle injuries: A matter of mechanism and age. *Accid. Anal. Prev*. **44**, 135–139 (2012). 10.1016/j.aap.2010.10.006 22062347

[33] Chen WS, Dunn RY, Chen AJ, Linakis JG (2013). Epidemiology of nonfatal bicycle injuries presenting to United States Emergency Departments, 2001–2008. Acad. Emerg. Med..

[34] Maya ST, Dena HJ, Kobi P (2012). Bicycle injuries: A matter of mechanism and age. Accid. Anal. Prev..

